# Analysis of *COCH* and *TNFA* Variants in East Indian Primary Open-Angle Glaucoma Patients

**DOI:** 10.1155/2013/937870

**Published:** 2013-08-26

**Authors:** Subhadip Chakraborty, Suddhasil Mookherjee, Abhijit Sen, Kunal Ray

**Affiliations:** ^1^Molecular & Human Genetics Division, CSIR-Indian Institute of Chemical Biology, 4 Raja S. C. Mullick Road, Kolkata 700 032, India; ^2^NEI/NIH, 6 Center Drive, Room 306 MSC 0610, Bethesda, MD 20892, USA; ^3^Dristi Pradip, 385 Jodhpur Park, Kolkata 700 068, India; ^4^Academy of Scientific and Innovative Research, Anusandhan Bhavan, 2 Rafi Marg, New Delhi 110 001, India

## Abstract

Glaucoma represents a heterogeneous group of optic neuropathies with a complex genetic basis. It is the second-largest cause of blindness in the world that reduces vision without warning and often without symptoms. Among 3 major subtypes of glaucoma, primary open-angle glaucoma (POAG) is the most common form. The focus of this study is to understand the molecular basis of the disease among Indian patients with respect to two genes, Cochlin (*COCH*) and tumor necrosis factor alpha (*TNFA*), selected based on reports of possible association with POAG. The genes were screened in patients and controls by PCR and direct sequencing. Although two novel changes (–450 C/T and –79 G/G) were identified in the 5′upstream region of *COCH*, no causal variant could be identified in either gene. –450 C/T was detected in 3 patients and 2 controls and –79 G/C in a single patient. Further, we did not observe significant association with the promoter SNPs of *TNFA* that had been previously reported to be associated with POAG pathogenesis. Thus, our study suggests lack of association of both *COCH* and *TNFA* with POAG pathogenesis.

## 1. Introduction

Glaucoma is a heterogeneous group of optic neuropathies characterized by typical visual field loss, often associated with increased intraocular pressure (IOP). It is the second leading cause of blindness after cataract and a silent killer of vision; often cases result in excessive loss of vision without any signs or symptoms. According to the latest estimate, 67 million people are visually impaired due to glaucoma worldwide, and among them 3.1 million are blind [1].

 Among different subtypes of glaucoma, primary open-angle glaucoma (POAG) is the most common form. POAG is a multifactorial complex disorder in which both environmental and genetic factors precipitate the disease. It has been suggested that 72% of POAG cases have some familial component, but on rare occasions the disease follow a Mendelian pattern of inheritance [2].

The complexity of POAG has been reviewed recently in detail [3–7]. There are 33 chromosomal loci that have been mapped through linkage analysis for POAG. However, only five underlying genes have been identified so far: *Myocilin* (GLC1A) [8] *Optineurin* (GLC1E) [9], *WD repeat domain 36* (GLC1G) [10], *NTF4* (GLC1O) [11], and *ASB10 *(GLC1F) [12]. In addition, candidate gene studies provide evidence for at least 30 POAG susceptibility genes. However, most studies demonstrate association in single population groups, and in some cases conflicting results have been reported in studies done on the same population. Therefore, it is difficult to judge whether the variations observed in these association studies are due to population difference, sample size, study design, or clinical heterogeneity between different cohorts of patients.

 Proteomic studies and expression analyses of the trabecular meshwork (TM) from patients with POAG and age-matched controls implicated Cochlin as a potential contributing factor to glaucoma pathogenesis [13–15]. Cochlin is a secretory extracellular matrix (ECM) protein of unknown function. Cochlin deposits were subsequently detected in glaucomatous, but not in control TM. Additionally, older glaucomatous TM was found to contain higher levels of Cochlin deposition. Cochlear Cochlin deposition is normally associated with an autosomal dominant nonsyndromic auditory and vestibular disorder (DFNA9) where misfolded Cochlin is thought to contribute to the formation of cochlear deposits. It was found that Cochlin deposits with mucopolysaccharide (MPS) develop in the TM of patients with POAG and glaucomatous DBA/2J mice [16]. This could potentially obstruct aqueous humor outflow and ultimately lead to elevated IOP. In both humans and mice, an age-related increase in Cochlin deposition was observed, which was consistently absent in normal TM tissues. 

Tumor necrosis factor-alpha (TNF-*α*) is a proinflammatory cytokine with multiple functions in the immune response. Since its initial discovery as a serum factor causing tumor necrosis [17], this potent immune-modulator has also been implicated in a wide spectrum of human diseases including sepsis, diabetes, cancer, collagen tissue diseases, and neurodegenerative diseases [18–20]. In recent years, it has been suggested that TNF-*α* may participate in apoptotic death of retinal ganglion cells in glaucoma patients [21, 22]. An upregulation of TNF-*α* and TNF-receptor 1 has also been observed in optic nerve heads and retinal sections of glaucomatous eyes [23]. Furthermore, an *in vitro *study has shown that glial cells exposed to elevated hydrostatic pressure or stimulated ischemia secrete increased amounts of TNF-*α*, subsequently leading to apoptotic death of cocultured retinal ganglion cells. This effect was attenuated by neutralizing antibodies against TNF-*α* [21]. Moreover, variations in the human *TNFA* gene promoter have been extensively studied for involvement in infectious diseases. SNPs at positions −238, −276, and −308 were analyzed for their possible association with diseases and their effects on promoter strength. The reported results are contradictory, and it is not yet clear if the SNPs under study are truly functional. To date, several reports have evaluated the association between *TNFA* promoter polymorphisms and risk for glaucoma. The results remain inconclusive [24–28]. However, such studies have not yet been undertaken in Indian POAG patients. 

With this background, we investigated the involvement of *COCH* and *TNFA* in glaucoma pathogenesis in a cohort of east Indian POAG patients. Both genes were screened in 100 patients and controls for any possible causal variants in the coding region. Additionally, 1 kb of the 5′ upstream region of *COCH* was screened. Two common pSNPs (−238 G/A and −308 G/A) in *TNFA* previously reported to be associated with POAG in different populations were also examined in a cohort of 285 patients and 285 controls.

## 2. Materials and Methods

### 2.1. Selection of the Study Subjects

The study cohort consisted of 285 POAG patients and 285 ethnically matched controls. East Indian patients affected with POAG, with or without a positive family history, were recruited in this study from the Dristi Pradip eye clinic (Kolkata, India). Diagnosis involved clinical, ocular, and systemic examinations. Ocular examinations involved measurement of IOP by applanation tonometry (Goldmann). Gonioscopy by Goldmann 3-mirror gonioscope (Shaffer's grading) revealed the angles of the anterior chamber and it was also used for optic disc evaluation and fundoscopy. Optic disc was further evaluated with +78D lens and visual field was assessed by Humphrey's automated perimeter. 

The POAG suspect was confirmed by typical reproducible visual field changes, namely arcuate, Bjerrum, Seidel, paracentral, and annular scotoma and nasal steps. In addition, scanning laser polarimetry for RNFL analysis (nerve fiber indicator >30) was also used to confirm the observation. An increased IOP above 21 mm of Hg, significant cupping of optic disc with or without peripapillary changes, and presence of an open angle of anterior chamber raised suspicion of POAG which was confirmed by typical reproducible visual field changes in automated perimetry test. Individuals with IOP less than 21 mm of Hg were also included, who had cupping of the optic disc and visual field changes characteristic of POAG. The IOP in each case was corrected for central corneal thickness (CCT). Individuals with any history of inflammation or ocular trauma (past and present) were excluded from this study. The age of patients at the time of diagnosis was >35 years (mean age ± SD, 60.49 ± 12.97 years).

The controls were recruited following a stringent set of criteria which include age > 40 years (mean age ± SD, 54.67 ± 11.27 years), without any family history of glaucoma or ocular hypertension, IOP < 20 mmHg in both eyes in at least the last two checkups, CCT greater than 500 *μ*m in both eyes, no visual field defect, normal scanning laser polarimeter parameters (i.e., a good yellowish bow type scan pattern, deviation map within normal limit, a good double hump pattern in conduction map), temporal-superior-nasal-inferior-temporal (TSNIT) parameters within normal limit, Nerve Fibre Indicator (<30 for both eyes), cup discs being physiological and similar in both eyes, cup to disc ratio <0.5, no defect in disc rim or margin, and no sphincter haemorrhage around the disc. Individuals with high myopia (>8 diopter), hypertension and diabetes were excluded from the control group. The study protocol adhered to the tenets of the declaration of Helsinki and was approved by the Institutional Review Board. 

### 2.2. Collection of Blood Samples and Genomic DNA Preparation

Eight milliliters of peripheral blood were collected with EDTA from the POAG patients and normal individuals with their written consent. Genomic DNA was prepared from fresh whole blood using the PAX gene blood DNA isolation kit (Qiagen, Hilden, Germany) according to the manufacturer's protocol. The DNA was dissolved in TE (10 mM Tris-HCl, 1 mM EDTA, pH 8.0). 

### 2.3. Polymerase Chain Reaction and DNA Sequencing

Screening was done in patients and controls by polymerase chain reaction (PCR) and sequencing. PCR was carried out in a total volume of 20.0 *μ*L containing 80 ng genomic DNA, 0.4 *μ*M of each primer, 10 *μ*L of 2x ex Prime Taq Premix (GeNet Bio, South Korea) with specific primers (primer sequences are available on request). Briefly, for *COCH*, each exon was amplified with an initial denaturation at 94°C for 5 min, followed by 35 cycles of denaturation at 94°C for 30 s, annealing at the range of 58–64°C for 30 s, and extension at 72°C for 30 s. A final extension at 72°C for 5 min completed the reaction. For *COCH* promoter amplification all the conditions were constant except the annealing temperature (at 52°C). For *TNFA*, being a small gene, all exons were amplified in a single PCR; briefly, an initial denaturation at 94°C for 5 min, followed by 35 cycles of denaturation at 94°C for 45 s, annealing at 68°C for 45 s, and extension at 72°C for 3 min. A final extension at 72°C for 5 min completed the reaction. For *TNFA* promoter SNPs [–308 G/A, (rs1800629) and –238 G/A, (rs361525)] genotyping, a segment of promoter was also amplified with an initial denaturation at 94°C for 5 min, followed by 35 cycles of denaturation at 94°C for 30 s, annealing at 53°C for 30 s, and extension at 72°C for 45 s. A final extension at 72°C for 5 min completed the reaction. All PCR products were detected on a 1.5% agarose gel with ethidium-bromide staining. Sequencing was done using the BigDye termination chemistry (version 3.1) in an ABI 3130XL capillary DNA sequencer (Applied Biosystems, Foster City, CA, USA) following manufacturer's protocol. 

### 2.4. Sequencing, Bioinformatics, and Statistical Analysis

The sequences obtained from the patients were compared with the wild type sequence by NCBI nucleotide blast online software (http://blast.ncbi.nlm.nih.gov/Blast.cgi). In addition, the chromatograms were critically examined for any “double peak” that would identify the heterozygous alleles. Haplotypes and their frequencies were calculated for comparison between patients and controls using Haploview 4.1 software (http://www.broad.mit.edu/mpg/haploview). The allele frequencies of the SNP and haplotypes were compared between patients and controls using contingency chi square test (95% CI). TFSEARCH (version 1.3) (http://www.cbrc.jp/research/db/TFSEARCH.html) was used to predict the transcription factor binding sites with default threshold score 85.0.

## 3. Results

**Figure 1:**
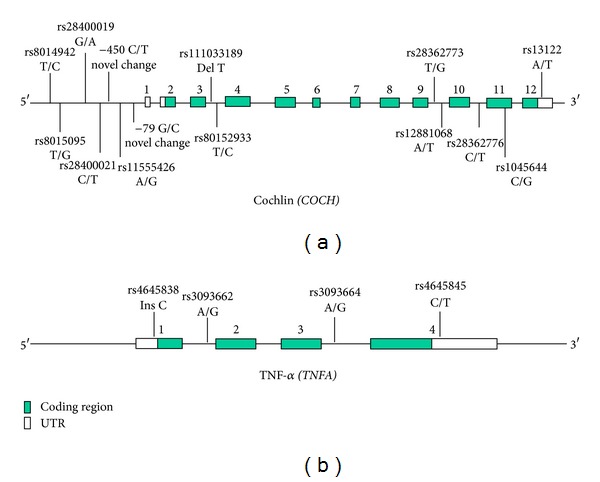
Schematic representation of *COCH* and *TNFA* genes with the location of SNPs and novel changes identified in the study. The sizes of exons and introns shown in the illustration are not according to scale.

**Figure 2:**
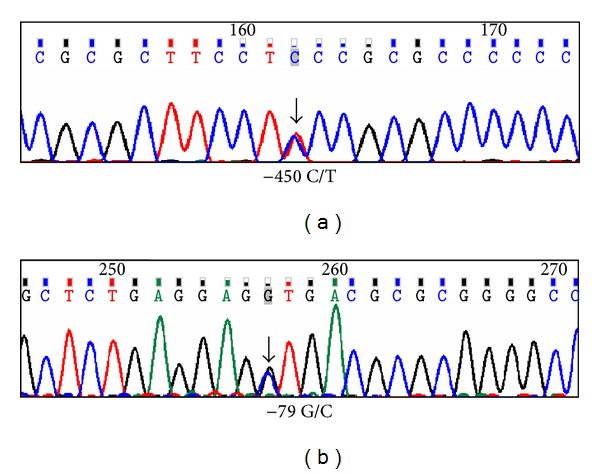
Representative chromatograms of the novel changes found in *COCH* screening. (a) −450 C/T change, found in three patients and two controls; (b) −79 G/C change, found in one patient only.

We screened all 12 exons, adjoining splice junctions and 1 kb of the upstream regulatory region of *COCH* in 100 POAG patients but did not find any variant in the gene that could be suspected as a mutation with the potential of causing POAG. Instead we detected a large number of SNPs, most of which are reported in dbSNP. These include 7 SNPs in the 5′ upstream region of the gene, 5 in the introns, 1 in exon 11 (Thr352Ser) and one in the 3′ UTR. Two of the SNPs (−450 C/T and −79 G/C) detected in the 5′ upstream region are novel (Figures 1 and 2). The frequencies of the SNPs in our cohort were different for some variants, which is likely due to differences in the populations genotyped (Table 1). The variant −450 C/T was detected in 3 patients and −79 G/C in a single patient. However, −450 C/T was also detected in 2 controls out of 100. *In silico* analysis of the novel changes revealed potential binding sites for multiple transacting factors. The −79 G/C change leads to abolition of CREB (cAMP response element-binding factor) binding site (TF search score 87.7), and −450 C/T leads to gain of a binding site for Sp1 (specificity protein 1) (TF search score 87.7) and E2F (TF search score 86.2). The other five reported SNPs in the 5′ upstream region of *COCH* (Table 1) did not reveal any changes in the binding sites of transacting elements except one (rs8015095, T/G), which leads to abolition of the GR (Glucocorticoid receptor) binding site (TF search score 85.4). However, all of this bioinformatics-derived information is subject to experimental verification. 

We detected only four SNPs in *TNFA*, located in the introns and UTRs, with frequencies different from those reported in dbSNP, possibly due to differences in the populations genotyped (Table 2). We genotyped two SNPs of *TNFA* [–308 G/A, (rs1800629) and –238 G/A, (rs361525)] that are reported to be associated with different disorders including POAG in our patient and control cohorts. However, no significant association was observed with either of the SNPs (*P* value > 0.05), neither in the allelic nor in the genotypic context (Tables 3 and 4). No significant association was found even after dividing the patients groups according to the presenting IOP (≥21 mm of Hg or <21 mm of Hg). None of the three different haplotypes, defined based on these two SNPs, showed any significant association with POAG (Table 5).

## 4. Discussion

In the present study, we screened the coding region of *COCH* and *TNFA *for the presence of disease causing mutations in East Indian POAG patient cohort. In addition, the 5′ upstream region of *COCH* was also analyzed for any potential disease causing regulatory variants. Two promoter SNPs (−308 G/A and −238 G/A) of *TNFA* were also genotyped in an additional 570 POAG patients and controls for their possible association with POAG. 

Although *COCH* mutations have only been associated with *DFNA9 *so far, a gene linked to hearing loss disorder [13–16], Cochlin deposits were detected in glaucomatous TM as well as DBA/2J glaucomatous mouse TM but not in normal controls [14]. Glaucomatous TM from older patients was also found to contain higher levels of Cochlin due to transcriptional upregulation [13, 29]. A recent report showed that overexpression and downregulation of Cochlin increases and decreases IOP, respectively, a potential risk factor of POAG [30]. Cochlin expression in glaucomatous TM (versus lack of expression in controls) suggests a potential release from “gene silencing” in the glaucomatous cases due to overexpression of other proteins. To understand the molecular basis of Cochlin expression in glaucomatous TM, we wanted to assess potential genetic variants in *COCH* in the POAG patient cohort, especially in the regulatory region of the gene. We did not find any mutations in the coding region of *COCH*. However, we did identify two novel variants in the 5′ upstream region of the *COCH* gene (−450 C/T and −79 G/C), which, upon *in silico* analysis, were found to alter the binding pattern of the transcription factors to the promoter region. These preliminary results are consistent with the reported alteration in *COCH* expression in the glaucomatous condition. In a similar study, Pertz et al. examined 190 glaucoma patients and did not find any potential disease causing variants in *COCH* except for one reported coding polymorphism (Thr352Ser), two synonymous variants (Phe389Phe and Asp423Asp), and an intronic variant (IVS4-8A > G). But, this study did not look for any potential regulatory region variants in the patient population [31].

Although the *in silico* analysis of the promoter variants identified in our study suggests a potential role in *COCH* expression, *in vitro* experiments using a reporter gene assay need to be executed to get unequivocal evidence to confirm the prediction. A detailed survey of functional promoter elements, followed by assignment of promoter strength with different variants, will be required to assess the true functional potential of genomic variants in the regulatory region of *COCH*. However, the overall effect of these variants on POAG pathogenesis is likely to be dependent on other factors that are yet to be learned through investigation.

Multiple studies to date have investigated the involvement of *TNFA *polymorphisms in POAG pathogenesis with variable outcomes [22, 24–28, 32–35]. We have analyzed the entire coding region of *TNFA* in our POAG patient cohort but failed to identify any disease causing mutation in this gene. In addition, two common promoter polymorphisms of *TNFA* (−238 G/A and −308 G/A) were genotyped in 570 POAG patients and controls. Both pSNPs were reported to influence TNF-*α* production, however the reports are controversial. Lipopolysaccharide- (LPS-) stimulated whole blood cell cultures from subjects with the −308 GA genotype were found to produce significantly higher levels of TNF-*α* compared to subjects carrying the −308 GG genotype [36, 37]. Also, the −238 GG genotype was reported to produce higher levels of TNF-*α* in LPS-stimulated whole blood cell cultures [38]. However, subsequent studies failed to replicate these observations [38–40]. Our study did not find any association of these two polymorphisms with the POAG patient cohort. It is worth mentioning here that although the −238 G/A polymorphism was never found to be associated with POAG, an increased prevalence of the *TNFA* −308 A allele is reported in POAG patient cohorts from China, Austria, Iran, and Turkey [24–26, 28, 32–35, 41]. However, a recent metaanalysis from 7 different reports from different populations did not identify any association of the −308 G/A SNP with POAG [28]. Interestingly POAG patients were found to have higher level of TNF-*α* in the aqueous humor of their eyes [42], but the question of whether it is a cause or an epiphenomenon of the disease still remains to be answered. 

## 5. Conclusions

To our knowledge, this is the first effort to ascertain the involvement of *COCH* and *TNFA* in an Indian POAG patient cohort. Our screening results suggested that *COCH* and *TNFA* genes are not associated with POAG pathogenesis in at least an East Indian POAG cohort. However, considering the minor contribution of each of the POAG loci identified so far to glaucoma pathogenesis, a screening effort in a much larger cohort from different regions of India could truly reflect the state of association of *COCH* and *TNFA* with POAG.

## Figures and Tables

**Table 1:** Variants identified in *COCH*.

SNP ID	Location	Minor allele frequency (db SNP*)	Minor allele frequency in patients
rs111033189 del T	Intron 3	NA	0.05
rs80152933 T/C	Intron 3	NA	0.05
rs28362773 T/G	Intron 9	0.138	0.025
rs12881068 A/T	Intron 9	0.145	0.045
rs28362776 C/T	Intron 10	0.022	0.04
rs1045644 C/G	Exon 11 (Thr352Ser)	0.467	0.15
rs13122 A/T	3′ UTR	0.175	0.01
rs8014942 C/T	5′ upstream region	0.4886	0.16
rs8015095 T/G	5′ upstream region	0.197	0.05
rs28400019 G/A	5′ upstream region	0.0847	0.03
rs28400021 C/T	5′ upstream region	0.2381	0.24
**−450 C/T ** **(novel change)**	5′ upstream region	NA	0.015 (0.01 in control)
rs11555426 G/A	5′ upstream region	0.10032	0.05
**−79 G/C ** **(novel change)**	5′ upstream region	NA	0.005 (not detected in control)

*http://www.ncbi.nlm.nih.gov/projects/SNP/.

**Table 2:** Variants identified in *TNFA*.

SNP ID	Location	Minor allele frequency (db SNP*)	Minor allele frequency in patients
rs4645838 ins C	5′ UTR	NA	0.02
rs3093662 A/G	Intron 1	0.079	0.01
rs3093664 A/G	Intron 3	0.075	0.005
rs4645845 C/T	3′ UTR	0.001	0.02

*http://www.ncbi.nlm.nih.gov/projects/SNP/.

**Table 3:** Allele frequency distribution of two SNPs of *TNFA* promoter in patients and controls.

SNP	Allele	Allele frequency in patients	Allele frequency in controls	*P* value	OR (95% CI)
rs361525 (−238 G/A)	G	0.897 (481)	0.912 (469)	0.406	1.192 (0.772–1.840)
	A	0.103 (55)	0.088 (45)	0.406	0.839 (0.543–1.295)

rs1800629 (−308 G/A)	G	0.957 (513)	0.955 (491)	0.884	1.045 (0.557–1.960)
	A	0.043 (23)	0.045 (23)	0.884	0.957 (0.510–1.796)

**Table 4:** Genotype frequency distribution of two SNPs of *TNFA* promoter in patients and controls.

SNP	Genotype	Genotypic frequency in patients	Genotypic frequency in controls	*P* value	OR (95% CI)
rs361525 (−238 G/A)	GG	0.81	0.833	0.567	0.855 (0.533–1.370)
	GA	0.175	0.16	0.712	1.120 (0.619–1.818)
	AA	0.015	0.007	0.686	1.932 (0.302–15.301)

rs1800629 (−308 G/A)	GG	0.918	0.911	0.876	1.099 (0.572–2.112)
	GA	0.078	0.089	0.753	0.865 (0.447–1.673)
	AA	0.004	0	—	—

**Table 5:** Haplotype frequency distribution of two SNPs of* TNFA *promoter in patients and controls.

Haplotype	Haplotype frequency in patients	Haplotype frequency in controls	*P* value	OR (95% CI)
G-G	0.857	0.868	0.72	0.923 (0.639–1.332)
G-A	0.1	0.088	0.458	1.197 (0.772–1.856)
A-G	0.04	0.045	0.763	0.874 (0.465–1.640)
